# Comprehensive 16S rRNA and metagenomic data from the gut microbiome of aging and rejuvenation mouse models

**DOI:** 10.1038/s41597-022-01308-3

**Published:** 2022-05-10

**Authors:** Jongoh Shin, Jung-Ran Noh, Donghui Choe, Namil Lee, Yoseb Song, Suhyung Cho, Eun-Jung Kang, Min-Jeong Go, Seok Kyun Ha, Jae-Hoon Kim, Yong-Hoon Kim, Kyoung-Shim Kim, Byoung-Chan Kim, Chul-Ho Lee, Byung-Kwan Cho

**Affiliations:** 1 Department of Biological Sciences and KI for the BioCentury, Korea Advanced Institute of Science and Technology, Daejeon, 34141 Republic of Korea; 2 Laboratory Animal Resource Center, Korea Research Institute of Bioscience and Biotechnology, Daejeon, 34141 Republic of Korea; 3 Immunotherapy Research Center, Korea Research Institute of Bioscience and Biotechnology, Daejeon, 34141 Republic of Korea; 4 Healthbiome Co., Ltd, Daejeon, 34141 Republic of Korea

**Keywords:** Metagenomics, Microbiome

## Abstract

The gut microbiota is associated with the health and longevity of the host. A few methods, such as fecal microbiota transplantation and oral administration of probiotics, have been applied to alter the gut microbiome and promote healthy aging. The changes in host microbiomes still remain poorly understood. Here, we characterized both the changes in gut microbial communities and their functional potential derived from colon samples in mouse models during aging. We achieved this through four procedures including co-housing, serum injection, parabiosis, and oral administration of *Akkermansia muciniphila* as probiotics using bacterial 16 S rRNA sequencing and shotgun metagenomic sequencing. The dataset comprised 16 S rRNA sequencing (36,249,200 paired-end reads, 107 sequencing data) and metagenomic sequencing data (307,194,369 paired-end reads, 109 sequencing data), characterizing the taxonomy of bacterial communities and their functional potential during aging and rejuvenation. The generated data expand the resources of the gut microbiome related to aging and rejuvenation and provide a useful dataset for research on developing therapeutic strategies to achieve healthy active aging.

## Abstract (summary)

**Table:** 

Measurement(s)	Microbiome
Technology Type(s)	Metagenome • Bacterial 16 S RNA
Factor Type(s)	Ageing • Co-housing • Parabiosis • Serum-injection • Akkermansia (AK) treatment
Sample Characteristic - Organism	Mus musculus
Sample Characteristic - Environment	animal cage
Sample Characteristic - Location	South Korea

## Background & Summary

The gut microbiota is associated with the health and longevity of the host. Through the aging process, age-related changes in the composition of gut microbiota have been observed^1–3^, which are related to increased intestinal disorders^4^, inflammation^5^, cognitive decline^6^, and increased frailty^1,7,8^. Furthermore, remodeling of the gut microbiome has resulted in a prolonged lifespan in *Drosophila melanogaster*^9,10^, killifish^11^, and progeroid mice^12^. Previous studies have clearly shown that delivery of a healthy microbiome through co-housing or fecal microbiota transplantation (FMT) enhances intestinal immunity and facilitates healthy aging^13–16^. In addition, heterochronic parabiosis allows young and old mice to be surgically combined to achieve remarkable regenerative effects in aged tissues^17–19^. Moreover, supplementation with probiotics changes the composition of microbes in the gut and improves gut immunity^11,12,16^. These studies have suggested that beneficial commensals (e.g., *Akkermansia*) can be used as a possible target to improve various age-related symptoms. Nevertheless, the aging process and changes in the gut microbiome by various rejuvenation methods have not yet been compared and described.

**Fig. 1:**
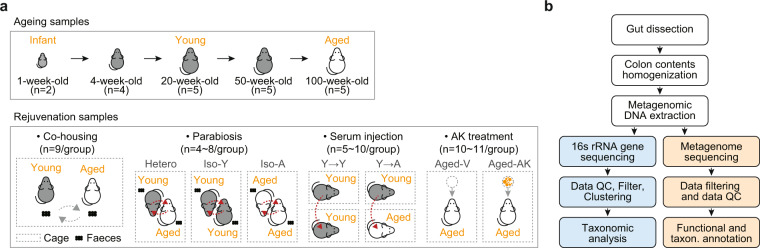
Overview of the experimental design. (**a**) Schematic description of the ageing model and four rejuvenation experiments including co-housing, parabiosis, serum injection, and AK treatment. (**b**) Workflow of data analysis. The analysis of 16 S rRNA data and metagenomic data are shown at the blue and yellow panel, respectively.

Here, we describe bacterial 16 S rRNA sequencing and metagenomic sequencing datasets derived from colon samples in mouse models during aging and four rejuvenation procedures including co-housing, serum injection, parabiosis, and oral administration of *Akkermansia muciniphila* as probiotics (Table 1). A diagram of the mouse models used in this study is shown in Fig. 1a. In the aging model, the time points of sample collection corresponded to five main phases of aging: week 1, week 4, week 20 (young phenotype), week 50, and week 100 (aged phenotype). For the co-housing model, young mice were co-caged with aged mice for six weeks in triplicate for three independent experiments. For the parabiosis model, the three parabiotic pairs were established as follows: (i) Hetero-Y (young mice from heterochronic pairs); (ii) Hetero-A (aged mice from heterochronic pairs) and Iso-Y (young mice from isochronic young pairs); and (iii) Iso-A (aged mice from isochronically aged pairs). For the serum injection model, aged (iv 8 Y → A and iv 16 Y → A) and young mice (iv 8 Y → Y and iv 16 Y → Y) were treated with serum collected from young mice by intravenous injection 8 times (for 3 weeks) or 16 times (for 6 weeks). Several blood parameters related to liver, kidney, and muscle function as well as lipids (Table 2), however, no significant changes in plasma markers were observed by parabiotic pairing. For the *Akkermansia* treatment model, the Aged-AK (*Akkermansia*-treated group) was treated daily with an oral administration of *Akkermansia* grown on a culture medium (BTTM) for 36 weeks and Aged-V (vehicle-treated group) was treated daily with BTTM only. These rejuvenation procedures restore age-dependent alterations in intestinal function and inflammation^20^. Furthermore, oral administration of *Akkermansia* led to an improvement in the frailty index (Supplementary Tables 1 and 2).

**Table 1:** Sample information in this study.

Library	Experiment	Condition	Sample #	Host	Material	Sequencing
16 S rRNA	Ageing	Week1	n = 2	C57BL/6 J	Colon	2 × 250 bp
		Week4	n = 4	C57BL/6 J	Colon	2 × 250 bp
		Week20 (Y)	n = 5	C57BL/6 J	Colon	2 × 250 bp
		Week50	n = 5	C57BL/6 J	Colon	2 × 250 bp
		Week100 (A)	n = 5	C57BL/6 J	Colon	2 × 250 bp
	Co-housing	Co-Y	n = 9	C57BL/6 J	Colon	2 × 250 bp
		Co-A	n = 9	C57BL/6 J	Colon	2 × 250 bp
	Parabiosis	Hetero-Y	n = 5	C57BL/6 J	Colon	2 × 250 bp
		Hetero-A	n = 4	C57BL/6 J	Colon	2 × 250 bp
		Iso-Y	n = 8	C57BL/6 J	Colon	2 × 250 bp
		Iso-A	n = 4	C57BL/6 J	Colon	2 × 250 bp
	Serum-injection	iv 8 (Y → A)	n = 9	C57BL/6 J	Colon	2 × 250 bp
		iv 16 (Y → A)	n = 5	C57BL/6 J	Colon	2 × 250 bp
		iv 8 (Y → Y)	n = 10	C57BL/6 J	Colon	2 × 250 bp
		iv 16 (Y → Y)	n = 4	C57BL/6 J	Colon	2 × 250 bp
	AK treatment	Aged-V	n = 10	C57BL/6 J	Colon	2 × 250 bp
		Aged-AK	n = 9	C57BL/6 J	Colon	2 × 250 bp
Shotgun metagenome	Ageing	Week1	n = 2	C57BL/6 J	Colon	2 × 250 bp
		Week4	n = 4	C57BL/6 J	Colon	2 × 250 bp
		Week20 (Y)	n = 5	C57BL/6 J	Colon	2 × 250 bp
		Week50	n = 5	C57BL/6 J	Colon	2 × 250 bp
		Week100 (A)	n = 5	C57BL/6 J	Colon	2 × 250 bp
	Co-housing	Co-Y	n = 9	C57BL/6 J	Colon	2 × 250 bp
		Co-A	n = 9	C57BL/6 J	Colon	2 × 250 bp
	Parabiosis	Hetero-Y	n = 4	C57BL/6 J	Colon	2 × 250 bp
		Hetero-A	n = 4	C57BL/6 J	Colon	2 × 250 bp
		Iso-Y	n = 8	C57BL/6 J	Colon	2 × 250 bp
		Iso-A	n = 4	C57BL/6 J	Colon	2 × 250 bp
	Serum-injection	iv 8 (Y → A)	n = 10	C57BL/6 J	Colon	2 × 250 bp
		iv 16 (Y → A)	n = 5	C57BL/6 J	Colon	2 × 250 bp
		iv 8 (Y → Y)	n = 10	C57BL/6 J	Colon	2 × 250 bp
		iv 16 (Y → Y)	n = 4	C57BL/6 J	Colon	2 × 250 bp
	AK treatment	Aged-V	n = 10	C57BL/6 J	Colon	2 × 250 bp
		Aged-AK	n = 11	C57BL/6 J	Colon	2 × 250 bp

**Table 2:** Blood parameters of parabiotic pairing.

	Blood parameters						
	AST	ALT	ALP	BUN	Crea	Chol	TG
	(IU/L)	(IU/L)	(IU/L)	(mg/dl)	(mg/dl)	(mg/dl)	(mg/dl)
Iso-Y	139 ± 11.8	45 ± 8.6	56 ± 5.3b	36 ± 5.2	0.2 ± 0.03a	122 ± 4.3ab	78 ± 16.1
Hetero-Y	123 ± 15.2	50 ± 8.5	65 ± 5.3ab	32 ± 2.1	0.2 ± 0.01a	113 ± 3.3b	81 ± 16.8
Hetero-O	116 ± 18.9	63 ± 11.6	73 ± 2.0a	39 ± 3.5	0.1 ± 0.02b	129 ± 6.0a	52 ± 6.0
Iso-O	157 ± 25.6	66 ± 10.7	72 ± 5.5a	32 ± 5.0	0.2 ± 0.03ab	100 ± 2.7	56 ± 3.8

Levels not connected by same letter (abc) are significantly different.

**Fig. 2:**
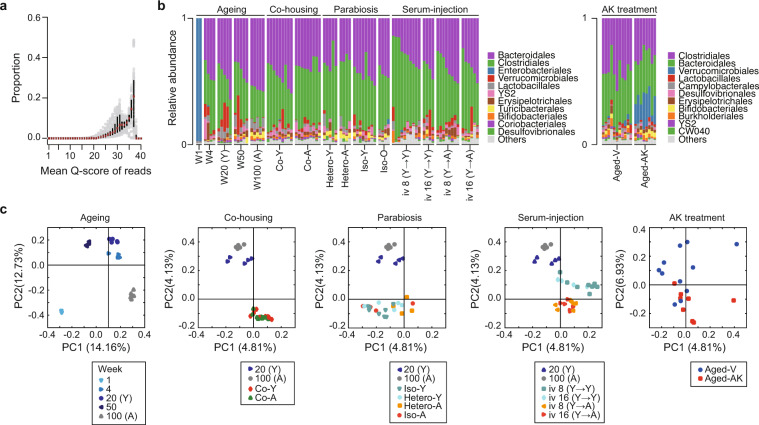
Overview of quality of 16 S rRNA libraries. (**a**) Dot plot showing read-quality of all 16 S rRNA libraries with a median and interquartile range of Phred quality score. (**b**) Bacterial community composition at the order level of colon samples analyzed in this study. Taxonomic assignments of the 11 most abundant taxa are given. The microbial profile shows dynamics with the underlying differential taxonomic abundance in the ageing and rejuvenation process. Taxonomic bar plots were generated using QIIME2. (**c**) Principal Coordinate Analysis (PCoA) of all datasets based on beta-diversity with Jaccard metric.

**Fig. 3:**
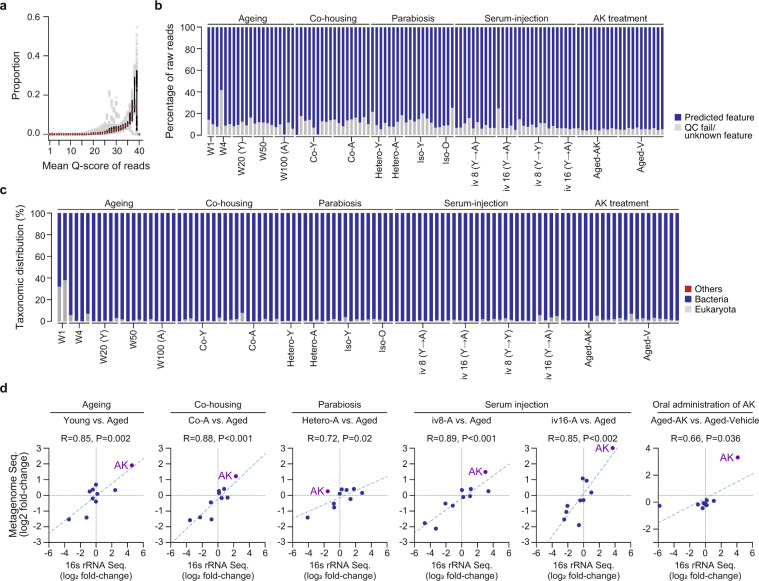
Overview of quality of metagenomic libraries. (**a**) Dot plot showing read-quality of all metagenomic libraries with a median and interquartile range of Phred -quality score. (**b**) The percentage of reads containing predicted features in raw reads after pair-merging, artefact removal, host DNA removal, and feature extraction. (**c**) The taxonomic prediction of raw reads is shown at the domain level. The “others” shown here means reads that contain the virus, archaea, unclassified taxa, and other sequences. (**d**) Correlation of taxonomic compositions (top 10) between 16 S rRNA sequencing and shotgun metagenomic sequencing. The relationship between the fold-change of taxonomic abundance from 16 S rRNA sequencing data and the fold-change of taxonomic abundance from metagenomic sequencing data was analyzed for top 10 taxonomic units at genus scale. “R” and “P” indicate the Pearson’s R and significance of the pairing, respectively. Significance was assessed by two-tailed *P*-values and we used an α level of 0.05 for all statistical tests. “AK” indicate the genus *Akkermansia*.

The raw sequencing data of 16 S rRNA sequencing and metagenomic sequencing comprised 36,249,200 paired-end reads generated from 107 samples and 307,194,369 paired-end reads generated from 109 samples (Supplementary Tables 3 and 4). The workflow of the data analysis is shown in Fig. 1b. The read-quality distribution of 16 S rRNA sequencing and metagenomic sequencing are shown in Fig. 2a and Fig. 3a, respectively. Operational taxonomic units (OTUs) were generated using DADA2^21^ in the QIIME2 pipeline^22^. The orders *Bacteroidales*, *Clostridiales*, *Enterobacteriales*, *Verrucomicrobiales*, and *Lactobacillales* dominated the bacterial communities (Fig. 2b). Based on beta-diversity analyses, microbial communities of colon contents from aging, co-housing, parabiosis, serum injection, and AK treatment experiments clearly separated (Fig. 2c), reflecting the small intra-group variation when compared to the inter-group variation. We employed the MG-RAST pipeline and predicted function of metagenomic data containing artefacts-removed, high-quality reads, a median of 91.1% predicted features per sample (range: 55.3%–99.6%), and a median of bacteria-driven reads per sample (range: 61.6%–99.8%) (Fig. 3b,c). The microbial functional enzyme abundance is shown in Dataset 1^23^. The classified bacterial taxonomy showed a significant positive correlation (Pearson’s R > 0.66, *P* < 0.036) between metagenomic sequencing data and 16 S rRNA sequencing data (Fig. 3d). Altogether, we enable reuse of the data and extend the collection of resources related to aging and rejuvenation related to the microbiome. The datasets described here offer an understanding of the relationship between aging and the gut microbiome and functional gene characterization of poorly known commensal bacteria related to aging. We anticipate that our data will pave the way for the development of therapeutic strategies to achieve healthy active aging.

## Methods

### Ethical statement

All animal experiments and animal care conditions were approved by the Institutional Animal Care and Use Committee (KRIBB-AEC-18211) and were performed in accordance with the institutional guidelines of the Korea Research Institute of Bioscience and Biotechnology.

### Ageing and rejuvenation mice models

We describe a more condensed version of the experimental methods for the construction of aging and rejuvenation mouse models in the primary publication^20^. A diagram of the mouse model used in this study is shown in Fig. 1a. C57BL/6 J mice were obtained from the Jackson Laboratory (Bar Harbor, ME, USA). These mice were housed at the Korea Research Institute of Bioscience and Biotechnology with a 12-h light/dark cycle with food and water ad libitum (Daejeon, Korea). For the co-housing experiment, three young mice (4 months old) were co-caged with three aged mice for six weeks (18 months old), and the co-housing experiments were performed in triplicate for three independent experiments (n = 9). For the parabiosis experiments, the were conducted as described previously^24^. The four parabiotic pairs were established as follows: (i) hetero-young (young mice from heterochronic pairs, n = 5); (ii) hetero-aged (aged mice from heterochronic pairs, n = 4); (iii) iso-young (young mice from isochronic young pairs, n = 6), and (iv) iso-aged (aged mice from isochronically aged pairs, n = 4). Effective blood circulation was assessed by intraperitoneally injecting 400 μL of 0.5% Evans blue dye into one of the parabionts^25^. For the serum injection experiments, serum was collected from young (4–5 months old) mice, pooled, and stored at −80 °C until use. Aged (20 months old; iv 8 times (iv 8 Y → A), n = 10; iv 16 times (iv 16 Y → A), n = 5) and young (5 months old; iv 8 times (iv 8 Y → Y), n = 10; iv 16 times (iv 16 Y → Y), n = 5) mice were treated with sera collected from young mice by intravenous injection 8 times (for 3 weeks) or 16 times (for 6 weeks). For AK treatment, aged mice (20–21 months old) were used. The *Akkermansia*-treated group (Aged-AK, n = 11) was treated daily with an oral administration of *Akkermansia* (4.9 × 10^8^ CFU per 150 µL per day) grown on a culture medium (BTTM) and the vehicle group (Aged-V, n = 10) was treated daily with BTTM only. Treatment was continued for 36 weeks.

*Akkermansia muciniphila* DSM 22959 T was obtained from the German Collection of Microorganisms and Cell Cultures (Leibniz-Institut DSMZ-Deutsche Sammlung von Mikroorganismen und Zellkulturen GmbH, Germany). *A. muciniphila* was grown anaerobically at 37 °C.

### Sample collection and DNA extraction

Mouse colon contents were collected, immediately frozen in liquid nitrogen, and stored at −80 °C. Total DNA from the snap-frozen colon content samples was extracted using the DNeasy PowerSoil DNA Pro Kit (Qiagen, Hilden, Germany) with a repeated bead-beating step for complete DNA extraction, as previously described^26^. The quantity and quality of the purified DNA were measured for 16 S rRNA and metagenomic sequencing using a Nanodrop spectrophotometer (Thermo Fisher Scientific, Massachusetts, USA), gel electrophoresis, and Qubit dsDNA HS Assay Kit (Thermo Scientific, Rockford, IL, USA).

### 16S ribosomal RNA (rRNA) and shotgun metagenomic sequencing

Targeted 16 S sequencing libraries were prepared according to the 16 S Metagenomics Sequencing Library Preparation protocol (Illumina, San Diego, CA, USA) in combination with the Illumina Nextera XT index and sequencing adapters. The protocol included two primers that selectively amplified the V3–V4 region-specific region of the 16 S gene. The resulting 16 s rRNA libraries were checked by agarose gel electrophoresis and an Agilent 2200 TapeStation (Agilent Technologies, Santa Clara, CA, USA) for the appropriate size. The concentration of 16 s rRNA libraries was assessed using the Qubit dsDNA HS Assay Kit on a Qubit 3.0 fluorometer and KAPA Library Quantification Kit for Illumina Platforms (Roche, Basel, Switzerland). Bacterial 16 S rRNA libraries were sequenced on a MiSeq and HiSeq. 2500 sequencing platform (Illumina, San Diego, CA, USA) using the dual index pair-end (2 × 250 bp) protocol, as recommended by the manufacturer. Metagenomic sequencing libraries were constructed using the Nextera XT DNA Library Preparation Kit (Illumina, San Diego, CA, USA) following the manufacturer’s recommendations. The resulting metagenomic sequencing libraries were checked using an Agilent 2200 TapeStation (Agilent Technologies, Santa Clara, CA, USA) for appropriate size. The concentration of metagenomic sequencing libraries was assessed using the Qubit dsDNA HS Assay Kit on a Qubit 3.0 fluorometer and KAPA Library Quantification Kit for Illumina Platforms (Roche, Basel, Switzerland). Sequencing was performed on a HiSeq. 2500 sequencing platform (Illumina, San Diego, CA, USA) using the dual index pair-end (2 × 250 bp) protocol, as recommended by the manufacturer.

### Microbial community profiling

Raw paired-end reads were processed using the QIIME 2 pipeline (ver. 2018.2.0) according to a previously described workflow^22^. Paired-end reads were trimmed and merged, and chimeric sequences were removed using the DADA2 plugin in QIIME2 (qiime dada2 denoise-paired)^21^. OTUs were clustered at 100% sequence similarity (amplicon sequence variants) using DADA2 and classified using the QIIME2 pre-trained naïve Bayes classifier trained on the Greengenes database (13_8). For phylogenetic diversity analyses, a phylogenetic tree was constructed (qiime phylogeny fasttree) by using an alignment of OTU sequence (qiime alignment “mafft” and “mask”). For beta diversity, pairwise permutational analysis of variance (PERMANOVA) statistics were calculated based on Jaccard using QIIME2 (qiime diversity core-metrics-phylogenetic). Principal coordinate analysis (PCoA) plots for beta diversity metrics were generated using the QIIME1 package (make_2d_plots.py).

### Metabolic function profiling

Using the CLC Genomics Workbench 6.5.1 (Qiagen), the raw reads were filtered and trimmed to remove low-quality bases and adaptor sequences. The FASTQ files of metagenomic data were uploaded to the Metagenomics Rapid Annotation using Subsystems Technology (MG-RAST) server version 4.0.3^27,28^. The resulting reads were trimmed and normalized, and sequencing artifacts were removed using the MG-RAST pipeline. Taxonomic assignment and annotation of metabolic functions of the metagenomic reads were performed using the non-redundant M5NR^29^ with a BLAT^30^ in the MG-RAST pipeline. Kyoto Encyclopedia of Genes and Genomes (KEGG) Orthology^31^, RefSeq, and SEED subsystem database tools^32^ were also employed for the alignment of functional genes, with default parameters^23^. The abundance of annotated genes and enrichment or depletion of bacterial taxonomic units were determined by read counts corresponding to the database.

## Data Records

We made the raw sequencing data available in the European Nucleotide Archive. Raw data of all 16 S rRNA sequences (fastq files) are available from the European Nucleotide Archive under project number PRJEB43096^33^. Raw data of all shotgun metagenomic sequencing data (fastq files) can be found at the European Nucleotide Archive under project number PRJEB43097^34^. Statistics for 16 S rRNA reads, statistics for metagenomic reads, and microbial functional enzyme abundance can be found in Supplementary 3, Supplementary Table 4, and Figshare^23^, respectively.

## Technical Validation

The 16 S rRNA libraries were checked by reviewing the absence of contamination by agarose gel electrophoresis and Agilent 2200 TapeStation. The 16 S rRNA sequencing and metagenomic raw reads were evaluated for their read qualities using the MultiQC^35^ wrapper for FastQC (Figs. 2a and 3a). The observed quality distribution showed that most of the reads scored Q25 or higher, indicating no quality problems. Quality control and denoising of 16 S rRNA sequencing reads were performed using the DADA2 plugin^21^ in the QIIME2 pipeline^22^. A clear difference in the microbial community structure was observed in the 16 S rRNA sequencing data from aging and four rejuvenation samples.

Raw DNA sequences from colon content for metagenome analysis showed sufficient bacterial sequences with any host DNA contamination (a median of 1.2% eukaryota reads), except for Week 1 samples in aging experiments (a median of 35.2% eukaryota reads) (Fig. 3c). All the metagenomic sequencing samples underwent data quality control to remove contaminants and trim reads to a Phred quality below 15. The bacterial taxonomy comparison between 16 S rRNA sequencing and metagenomic sequencing data demonstrated the strong consistency of the bacterial microbiome structure on aging and four rejuvenation experiments (Fig. 3d).

## Supplementary information

Supplementary Table 1. Frailty scores and clinical frailty index in Aged-Vehicle samples at 0 and 30 weeks.

Supplementary Table 2. Frailty scores and clinical frailty index in Aged-AK samples at 0 and 30 weeks.

Supplementary Table 3. Read statistics of 16s rRNA data.

Supplementary Table 4. Read statistics of metagenome data.

## Data Availability

In this analysis, default parameters or parameters recommended by the developer were used. The options used for the processing of the 16 S rRNA sequencing were as follows:qiime dada2 denoise-paired ––i-demultiplexed-seqsdemux-paired-end.qza ––o-table table ––o-representative-sequences rep-seqs ––p-trim-left-f 6–-p–trim-left-r 6 ––p-trunc-len-f 250 ––p-trunc-len-r 250. The options used for processing the metagenomic data in the CLC Genomics Workbench (ver. 6.5.1) is as follows:trimming: limit = 0.05, maximum two ambiguousnucleotides allowed

## References

[1] Claesson MJ (2012). Gut microbiota composition correlates with diet and health in the elderly. Nature.

[2] Claesson MJ (2011). Composition, variability, and temporal stability of the intestinal microbiota of the elderly. Proc National Acad Sci.

[3] Buford TW (2017). Dis)Trust your gut: the gut microbiome in age-related inflammation, health, and disease. Microbiome.

[4] Kolling G, Wu M, Guerrant RL (2012). Enteric pathogens through life stages. Front Cell Infect Mi.

[5] Fransen F (2017). Aged Gut Microbiota Contributes to Systemical Inflammaging after Transfer to Germ-Free Mice. Front Immunol.

[6] Cattaneo A (2017). Association of brain amyloidosis with pro-inflammatory gut bacterial taxa and peripheral inflammation markers in cognitively impaired elderly. Neurobiol Aging.

[7] Rampelli S (2013). Functional metagenomic profiling of intestinal microbiome in extreme ageing. Aging.

[8] Biagi E (2010). Through Ageing, and Beyond: Gut Microbiota and Inflammatory Status in Seniors and Centenarians. Plos One.

[9] Westfall S, Lomis N, Prakash S (2018). Longevity extension in Drosophila through gut-brain communication. Sci Rep-uk.

[10] Obata F, Fons CO, Gould AP (2018). Early-life exposure to low-dose oxidants can increase longevity via microbiome remodelling in Drosophila. Nat Commun.

[11] Sonowal R (2017). Indoles from commensal bacteria extend healthspan. Proc National Acad Sci.

[12] Bárcena C (2019). Healthspan and lifespan extension by fecal microbiota transplantation into progeroid mice. Nat Med.

[13] Ma J (2020). Gut microbiota remodeling reverses aging-associated inflammation and dysregulation of systemic bile acid homeostasis in mice sex-specifically. Gut Microbes.

[14] Shin JH (2017). Innate Immune Response and Outcome of Clostridium difficile Infection Are Dependent on Fecal Bacterial Composition in the Aged Host. J Infect Dis.

[15] Gupta S, Allen-Vercoe E, Petrof EO (2016). Fecal microbiota transplantation: in perspective. Ther Adv Gastroenter.

[16] Stebegg M (2019). Heterochronic faecal transplantation boosts gut germinal centres in aged mice. Nat Commun.

[17] Baht GS (2015). Exposure to a youthful circulation rejuvenates bone repair through modulation of β-catenin. Nat Commun.

[18] Conboy IM (2005). Rejuvenation of aged progenitor cells by exposure to a young systemic environment. Nature.

[19] Villeda SA (2014). Young blood reverses age-related impairments in cognitive function and synaptic plasticity in mice. Nat Med.

[20] Shin J (2021). Ageing and rejuvenation models reveal changes in key microbial communities associated with healthy ageing. Microbiome.

[21] Callahan BJ (2016). DADA2: High-resolution sample inference from Illumina amplicon data. Nat Methods.

[22] Caporaso JG (2010). QIIME allows analysis of high-throughput community sequencing data. Nat Methods.

[23] Shin J (2021). Figshare.

[24] Kamran, P. *et al*. Parabiosis in Mice: A Detailed Protocol. *J Vis Exp*10.3791/50556 (2013). 10.3791/50556 PMC3938334 24145664

[25] Mackie WS (1976). Plasma volume measurements in sheep using Evans’ blue and continuous blood sampling. Res Vet Sci.

[26] Shin J (2016). Analysis of the mouse gut microbiome using full-length 16S rRNA amplicon sequencing. Sci Rep-uk.

[27] Meyer F (2008). The metagenomics RAST server – a public resource for the automatic phylogenetic and functional analysis of metagenomes. Bmc Bioinformatics.

[28] Meyer F (2017). MG-RAST version 4—lessons learned from a decade of low-budget ultra-high-throughput metagenome analysis. Brief Bioinform.

[29] Wilke A (2012). The M5nr: a novel non-redundant database containing protein sequences and annotations from multiple sources and associated tools. Bmc Bioinformatics.

[30] Kent WJ (2002). BLAT—The BLAST-Like Alignment Tool. Genome Res.

[31] Kanehisa M, Sato Y, Kawashima M, Furumichi M, Tanabe M (2016). KEGG as a reference resource for gene and protein annotation. Nucleic Acids Res.

[32] Overbeek R (2014). The SEED and the Rapid Annotation of microbial genomes using Subsystems Technology (RAST). Nucleic Acids Res.

[33] (2021). European Nucleotide Archive.

[34] (2021). European Nucleotide Archive.

[35] Ewels P, Magnusson M, Lundin S, Käller M (2016). MultiQC: summarize analysis results for multiple tools and samples in a single report. Bioinformatics.

